# The Role of Omega-3 Fatty Acids in the Treatment of Depression in Children and Adolescents: A Literature Review

**DOI:** 10.7759/cureus.44584

**Published:** 2023-09-02

**Authors:** Tanu Thakur, Sukhmanjeet Kaur Mann, Narpinder Kaur Malhi, Raman Marwaha

**Affiliations:** 1 Psychiatry, MetroHealth Medical Center Case Western Reserve University, Ohio, USA; 2 Psychiatry, Sri Guru Ram Das Institute of Medical Sciences & Research, Sri Amritsar, Amritsar, IND; 3 Child and Adolescent Psychiatry, ChristianaCare, Delaware, USA; 4 Child and Adolescent Psychiatry, MetroHealth Medical Center Case Western Reserve University, Ohio, USA

**Keywords:** child and adolescent, omega-6/omega-3 ratio, eicosapentaenoic acid, docosahexaenoic acid, depressive disorder, omega-3 fatty acid

## Abstract

Depression is one of the most common mental disorders diagnosed in children and adolescents. Many individuals benefit from pharmacotherapy including antidepressants, however, there is a fair likelihood of remission and recurrence. Of the several pathophysiologies, depression has been linked to inflammation. Complementary and alternative medications such as the use of omega-3 fatty acids are gaining popularity given their anti-inflammatory properties. The goal of this literature review is to assess the efficacy and the clinical use of omega-3 fatty acids in children and adolescents with depression. We conducted an extensive literature search on PubMed, Ovid MEDLINE, and PsycINFO from January 1, 2005, to September 2021, for published articles (case reports, systematic review, RCT) in any language. A total of seven published studies were included in our literature review. Results indicated a huge heterogenicity in the studies and hence the clinical use of omega-3 fatty acids as monotherapy in depression was not determined. However, it was well tolerated with an extremely low side effect profile. Further research on the use of omega-3 fatty acids as an adjunct to antidepressants would be valuable.

## Introduction and background

Depression is one of the most common mental disorders diagnosed in children. Around 3.2% of children aged 3 to 17 years (approximately 1.9 million) are diagnosed with depression each year [1]. Although most episodes of depression remit within a year, the risk of recurrence is as high as 50-70% within the next five years [2]. Moreover, 40% of adolescents fail to respond to initial pharmacotherapy [3] and less than a third of adolescents reach remission with the initial treatment [4]. With the high likelihood of remission and recurrence, there is a higher risk of suicide. Suicide is among the leading causes of death among ages 10 to 24 [5].

Various theories regarding the pathophysiology of depression have been determined. One suggested theory for depression pertains to inflammation. Multiple clinical trials and meta-analyses have proven to identify a positive correlation between increased levels of C reactive protein, interleukin 6, interleukin 1, and tumor necrosis factor-alpha with major depressive disorder [6]. Inflammation can impact neuroplasticity and decrease neurogenesis, eventually leading to increased glutamatergic activation and thereby causing oxidative stress and induction of apoptosis [7].

One of the most popularly studied medications includes omega-3 fatty acids, which have shown promising results. Omega-3 fatty acids were discovered in 1929 by George Burr and Mildred Burr [8] with extensive research ranging from their role in cardiovascular risk to exploring their roles in neuropsychiatric pathological processes [9]. Omega-3 fatty acid precursor, alpha-linolenic acid (ALA) like omega-6 precursor, linoleic acid (LA), is an essential fatty acid not synthesized by the body. Omega-3 fatty acids are unsaturated fatty acids that constitute 35% of lipids in the brain [10]. Omega-3 fatty acids such as eicosapentaenoic acid (EPA) and docosahexaenoic acid (DHA) are derived from ALA [11]. Arachidonic acid (AHA) and DHA are the main omega-6 and omega-3 fatty acids. Primary sources of omega-3 fatty acids are found in eggs, milk, and vegetables.

EPA and DHA have several important anti-inflammatory properties including decreased chemotaxis of neutrophils and monocytes, decreased expression of adhesion molecules on the surface of immune cells and in the circulation, decreased production of prostaglandins, and inhibition of T-cell proliferation [12]. Omega-3 fatty acids also reduce the synthesis of proinflammatory mediators such as interleukin-1B and tumor necrosis factor- α by competitive inhibition of omega-6 fatty acids [13]. In fact, in Western society, the dietary intake of omega-6 sharply exceeds that of omega-3 which may promote a state of systemic low-grade inflammation [14]. It has been proven that a high omega-6 to omega-3 ratio, especially if above 9:1, is associated with an increased risk of postpartum depression [15].

Omega-3 fatty acids are also required by the neural network for increased connectivity and faster neurotransmission. They are vital for the stabilization and conformation of Na⁺/K⁺-ATPase (sodium and potassium ATPase), calcium channels, and sodium and chloride ion channels, thus maintaining neural membrane potential and transmission [13]. A deficiency of these fatty acids can lead to neuron hypo-functioning and eventually neuron death [13].

The ratio of DHA to other fatty acids in the brain affects membrane fluidity and the functioning of enzymes, including tyrosine hydroxylase, the rate-limiting enzyme in dopamine synthesis [16], ion channels, and receptor binding affinity and expression. Omega-3 fatty acids have been shown to affect serotonin and dopamine neurotransmission by altering phospholipid composition thereby modifying the structural and functional membrane proteins [17,18]. Omega-3 fatty acids are significant in the maintenance of membrane integrity for the serotonergic type 2 receptors in the prefrontal cortex and tryptophan cellular transport. It is responsible for preventing oxidative stress and inflammation of serotoninergic neurons and thereby maintaining mood state [13]. Omega-3 fatty acids also affect neuroplasticity and cell survival through neurotrophins such as brain-derived neurotrophic factor (BDNF) [19]. Furthermore, omega-3 concentrations have also been found to affect gene expression [19]. Additionally, studies have shown that low levels of EPA and/or DHA have been noted in social anxiety disorder [20] and bipolar disorder [21]. Red blood cell fatty acid levels have also been found to be lower in depressed adolescents [22].

In this review, we examine the randomized controlled trials done from year January 2005 to September 2021 to determine the effect and clinical use of omega-3 fatty acids in the treatment of depression in children and adolescents.

## Review

Methods

The purpose of this review was to determine the effect and the clinical use of omega-3 fatty acids in the treatment of depression in children and adolescents. We conducted an extensive literature search on PubMed, Ovid MEDLINE, and PsycINFO, for published articles (case reports, systematic review, RCT) in any language from January 1, 2005, to September 2021. Keywords were as follows: omega-3 fatty acids, omega-6 fatty acids, depression, children, adolescents, and mixed anxiety depressive disorder (MADD). We also reviewed the bibliographic databases of published articles for additional studies.

Inclusion and exclusion criteria were developed to pilot the search and selection criterion. A total of 811 articles were identified and after removing the duplicates, 236 articles were screened. It was narrowed down to seven published studies that were included in our literature review with ages ranging between 6 and 19 years. Inclusion criteria were clinical trials, case reports, and systematic reviews conducted for the use of omega-3 fatty acids in children and adolescents under 18 years of age suffering from depressive disorder (DD) and mixed anxiety depressive disorder (MADD). The objective scales used to measure the improvement in depressive symptoms involving only human subjects were included, that were published in English-language or had an official English translation. The studies that didn't fall between the age desired for review or didn't include the keywords were set for exclusion. Therefore, exclusion criteria included subjects above the age of 18, participants with unstable physical conditions, psychotic disorders, bipolar disorders, somatic disorders, personality disorders, organic mental disorders, substance use, and eating disorders. 

**Figure 1:**
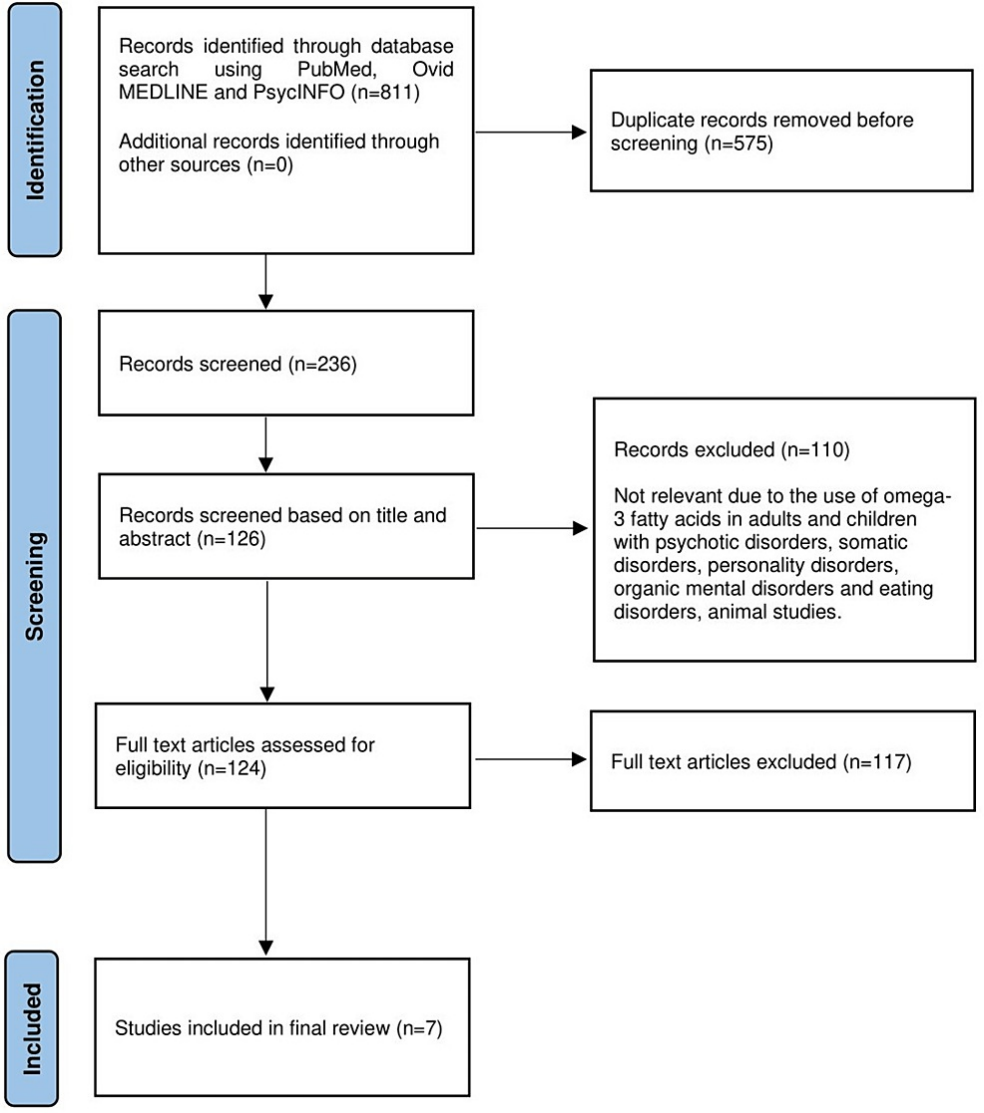
Flowchart depiction (PRISMA diagram) of the literature review selection process PRISMA, Preferred Reporting Items for Systematic Reviews and Meta-Analyses

The abstracts and the full-text articles from the citations considered through the database search were reviewed by all the authors and a consensus was reached for the final analysis of studies that would be included in the review. A systematic review was piloted in agreement with the directions of PRISMA (Preferred Reporting Items for Systematic Review and Meta-analyses) (Figure 1).

Mechanism of action of omega-3 fatty acids

Omega-3 fatty acids contribute to one-fifth of the brain’s dry weight with 33% of fatty acids belonging to the polyunsaturated fatty acids (PUFA) class [23,24]. Omega-3 fatty acids are responsible for the stabilization and conformation of receptors and structural ligands, a lack of which can alter cell membrane functioning and affect signal transmission. This could further affect the serotonergic activity in the prefrontal, parietal, and somatosensory cortex [25]. Their activation of the glutamatergic system via the L-type CA2+ channel/glutamate receptor subunit N-methyl D-aspartate A2B/voltage-dependent anion channel signaling prevents neuronal cell death [26]. Lower omega-3 fatty acids increase tissue inflammation leading to lower levels of cAMP response element binding factor, and brain-derived neurotrophic factor, affecting synaptic plasticity and accelerating neuronal cell death [27].

Several studies have discovered reduced omega-3 levels in depressed individuals. Moreover, the EPA content in red blood corpuscles (RBC) phospholipids is inversely proportional to the severity of depression and the omega-6 AA-to-EPA ratio was directly proportional to the clinical symptoms of depression [28]. Interestingly, suicide attempts have been correlated to low levels of RBC EPA. Additionally, evidence points towards PUFA levels varying seasonally, with peaks for EPA and DHA from August to September [29]. Despite all this evidence, it is important to note that not every study supports and has found an association between lowered omega-3 status and depression.

Long chain (LC)-PUFA biosynthesis is regulated by fatty acid delta-6 desaturase (FADS2) and fatty acid delta-5 desaturase (FADS1) as well as elongases (e.g., ELOVL5) [30]. Desaturase enzymes are in turn regulated by several factors such as gonadal hormones [31], insulin [32], as well as SNPs within FADS1 and FADS2. Recent evidence further suggests that epigenetic factors are associated with delta-5/delta-6 desaturase enzyme activity. Evidence from genome-wide association studies supports DNA methylation in the ELOVL5 gene being associated with depression and suicidality [31] but no association was found with FADS2 and FASD1 genes. However, FADS1 mRNA expression was significantly lower in the postmortem prefrontal cortex of depressed individuals relative to controls and there were trends for lower expression of FADS2 and ELOVL5 [33]. Cross-sectional studies in pediatric and adolescent populations with major depression exhibited erythrocyte EPA and DHA deficits compared to healthy controls [34]. DHA deficits were also observed in the anterior cingulate and the prefrontal cortex of adult patients with MDD [35].

Recent neuroimaging studies in children found low erythrocyte DHA associated with reduced functional connectivity within the prefrontal cortical networks [36]. Studies have shown treatment efficacy of supplementation with omega-3 PUFA in depression is influenced by the proportion and dosage of EPA or DHA with EPA >50, 60, and 80% of the dose [37-39] with the ratio of EPA and DHA of 2:1 or 3:1 also with DHA 1g/d or 2g/d were more efficacious than 4g/d [38].

Results

The seven randomized controlled trials included children with a diagnosis of depression, dysthymia, mixed anxiety depression (MADD), or unspecified depression. These studies were conducted from years from 2005 to 2021. Measures commonly used to assess depression included CDRS-R (Children’s Depression Rating Scale-Revised), (Children’s Depression Rating Scale) CDRS, CDI (The Children’s Depression Inventory), CGI (Clinical Global Impression), CES-D (Center for Epidemiologic Studies Depression Scale) and BDI (Beck’s Depression Inventory). Dietary intake of omega-3 fatty acids was taken into consideration in the studies. A comparison of the study designs of each RCT is drawn as seen in Table 1.

**Table 1:** Summary depicting outcome of the reviewed studies CDRS = Children's Depression Rating Scale; CDRS-R = Children's Depression Rating Scale, Revised; CDI = Children's Depression Inventory; CGI = Clinical Global Impressions Scale; CES-D = Center for Epidemiologic Studies Depression Scale; BDI = Beck's Depression Inventory

Studies	No. of Patients	No. of patients who dropped out	Age range	Duration of treatment	The scale used to measure	Outcome	Side- effects
Nemets et al., 2006 [39]	20 (10 in omega-3 and 10 in placebo)	8	6-12 years	16 weeks	CDRS, CDI, CGI	7/10 had a greater than 50% reduction in CDRS scores and showed statistical significance	None
Arnold et al., 2017 [40]	64 (28 in omega-3 and 34 in placebo)	8	7-14 years	12 weeks	CDRS-R	CRDS-R score showed significant improvement in the combined PEP and omega-3 group but not in the other groups	None
Trebaticka et al., 2017 [41]	35 (17 in omega-3 and 18 in omega-6 placebo)	3	7-18 years	16 weeks	CDI	CDI score was reduced in the omega-3 group from 24.4 to 25.5% which showed s statistical significance	None
Trebaticka et al., 2020 [42]	58 (29 in omega-3 and 29 in omega-6 placebo)	2	7-18 years	16 weeks	CDI	CDI score in the Om3 group was observed after 10 weeks and showed the highest reduction from −7.6, −27.4% of baseline score which showed statistical significance	1/29 in the omega-3 group had frequent defecation (2–3x daily).
Fristad et al., 2016 [43]	69	15	7-14 years	12 weeks	CDRS-R	No significant improvement	1 or no S/E out of constipation, diarrhea, stomach-ache, increased/decreased appetite, burping, fishy breath, nausea
Gabbay et al., 2019 [44]	39 (21 in omega-3 and 13 in placebo)	12	12-19 years	10 weeks	CDRS-R, BDI, Beck Scale for suicidal ideations	CDRS-R score of ≤28 which meant no significant improvement	None
Van der Wurff et al., 2020 [45]	256 at baseline; 199 at 12 months (95 krill oil, 104 placebo)	57	Second-year high-school students (14-18 years)	12 months	CES-D	Not significant statistically	None

A total of 541 patients were studied in the seven studies aforementioned. We found that four of the seven studies, those by Nemets et al. [39], Arnold et al. [40], Trebaticka et al. (2017) [41], and Trebaticka et al. (2020) [42], to have statistically significant improvement in depression scales. However, the scales used to measure were variable, and the dosage ratio in which eicosapentaenoic acid (EPA) and docosahexaenoic acid (DHA) were given also varied. Omega-3 fatty acids were tolerated well by most of the patients. Compliance was monitored by pill counts from the returned pill boxes or via the guardians doing a capsule count.

Despite the significant success of omega-3 fatty acids in depression in the studies, the other three studies, those by Fristad et al. 2016 [43], Gabbay et al. [44], and Van Wurff et al. [45], found no appreciable results, thereby contributing to the mixed results of our analysis.

Discussion

The current literature has seven randomized controlled trials conducted in children and adolescents. Looking at the above studies and reviewing those shows mixed results of using omega-3 fatty acids. However, it is also important to note that there is a huge heterogeneity in study designs. Although all studies were randomized controlled trials, the inclusion criteria were not uniform. At first, the diagnostic criteria for depression varied. Some studies have defined depression based on the DSM IV criteria; others based on ICD-10. Nemets et al. used the childhood version of the Schedule for Affective Disorders and Schizophrenia for major depressive disorder. Moreover, although the age groups ranged under 18 years, some studies included participants who were either adolescents or younger children. Furthermore, studies like Trebaticka et al. included participants using antidepressants while other studies such as Arnold et al. did not include participants undergoing pharmacotherapy or psychotherapy. Fristad et al. studied omega-3 treatment with and without psychotherapy. Next, assessment scales differed. Some studies used the CDRS or CDRS-R assessment while some studies used CDI or self-assessment scales. Self-rating questionnaires could lead to false positives or false negatives. Additionally, omega-3 dosage and timing of administration varied. Also, some studies measured the external food sources of omega-3 fatty acids consumed by the study groups; however, some studies did not. Lastly, the control groups differed in the studies as some received sunflower oil, while others received olive oil. The different references have possibly various modulating compounds and content.

It is important to highlight the strengths of the studies. The studies did take into consideration excluding participants with an inability to swallow capsules. The studies were randomized controlled trials that were carefully constructed with more statistical reliability. Baseline assessments were done and variables such as age, weight, sex, and treatment history were taken into consideration. Studies such as the one by van der Wurff et al. collected blood samples to monitor omega-3 levels in the blood to assess compliance, including EPA and DHA levels on every assessment. Participants with medical issues were excluded ruling out any malabsorption syndromes.

This review has several limitations. To begin, the studies included were limited by their small sample sizes. Next, the timing and instructions for the administration of omega-3 fatty acids were not clear to participants. This could have affected the absorption of omega-3 fatty acids which are better absorbed with fatty food. Finally, most of the studies were limited to 12 weeks which limits our understanding of the pharmacodynamics of omega-3 fatty acids.

Nevertheless, we learned new information from the RCTs regarding the practicality of recommending omega-3 fatty acids to children and adolescents. Omega-3 fatty acids have not shown any clear benefit as monotherapy for depression.

## Conclusions

Depression has a high incidence in children and adolescents and is associated with impairment in social and occupational functioning, debilitating when severe. Pharmacotherapy consisting of antidepressants is not always effective for the general population groups. Alternative medications are hence gaining popularity. Our review shows the utility of omega-3 supplementation in mitigating depression in children and adolescents and shows mixed results. Due to the heterogenicity in our results, it is insufficient to draw solid conclusions to show their superiority as a monotherapy over antidepressants. Further research requires a larger database and more uniformity in study designs to establish the use of omega-3 fatty acids for the treatment of depression in children and adolescents.
